# ﻿Taxonomic study on the genus *Mongoloniscus* Verhoeff, 1930 (Isopoda, Agnaridae) from China: morphological and phylogenetic analyses

**DOI:** 10.3897/zookeys.1202.113560

**Published:** 2024-05-23

**Authors:** Chao Jiang, Jing Zhong, Zhidong Wang, Weichun Li, Luqi Huang

**Affiliations:** 1 State Key Laboratory for Quality Ensurance and Sustainable Use of Dao-di Herbs, National Resource Center for Chinese Materia Medica, China Academy of Chinese Medical Sciences, Beijing 100700, China China Academy of Chinese Medical Sciences Beijing China; 2 College of Agronomy, Jiangxi Agricultural University, Nanchang 330045, China Jiangxi Agricultural University Nanchang China

**Keywords:** DNA, morphology, new species, Oniscidea, taxonomy

## Abstract

A combination of morphological traits and DNA data (COI and 28S rRNA partial sequences) was used to study the genus *Mongoloniscus* Verhoeff, 1930 from China. Four new species are described: *M.crenatus* Jiang, Li & Huang, **sp. nov.**, *M.orientalis* Jiang, Li & Huang, **sp. nov.**, *M.polyacanthum* Jiang, Li & Huang, **sp. nov.**, and *M.parvus* Jiang, Li & Huang, **sp. nov.** Following an in-depth examination of the *Mongoloniscus* species, *Lucasioidesvannamei* (Arcangeli, 1927), **comb. nov.** (from *Mongoloniscus*) is proposed, and *M.chevronus* Yang & An, 2021, **syn. nov**. is synonymized with *Koreoniscusracovitzai* (Arcangeli, 1927). A restrictive criterion for recognizing the genus *Mongoloniscus* is also provided in the present study.

## ﻿Introduction

Agnaridae, one of 38 families within the order Oniscidea worldwide, comprises 14 genera. At present, six genera, namely *Hemilepistus* Budde-Lund, 1879, *Agnara* Budde-Lund, 1908, *Protracheoniscus* Verhoeff, 1917, *Mongoloniscus* Verhoeff, 1930, *Koreoniscus* Verhoeff, 1937, and *Lucasioides* Kwon, 1993 have been recorded (Kwon 1993; Kwon and Taiti 1993; Tang and Gui 2000; Li 2017; Chen 2003; Wang et al. 2022a, 2023). However, taxonomic research on the Chinese woodlice is far from complete. In the present study, we focused on the taxonomy of *Mongoloniscus* from China.

*Mongoloniscus* was established by Verhoeff (1930) as a subgenus of *Protracheoniscus* Verhoeff, 1917 with P. (M.) koreanus Verhoeff, 1930 proposed as its type species. To date, this genus consists of eighteen species. All known species are recorded from East Asia (China, Korea, and Japan) (Schmalfuss 2003; Boyko et al. 2008; Nunomura 2010a, 2010b, 2013; Li 2017) except *M.persicus* Kashani, 2014, which has been reported in Iran (Kashani 2014). Four *Mongoloniscus* spp. have been recorded in China. Among them, *M.sinensis* (Dollfus, 1901) and *M.chevronus* Yang & An, 2021 are endemic to northern China (Chen 2003; Zhao et al. 2016; Yang and An 2021), whereas *M.koreanus* (Verhoeff, 1930) and *M.vannamei* (Arcangeli, 1927) are not only found across several southern Chinese provinces (Kwon 1993; Kwon and Taiti 1993; Chen 2003) but also have been reported in Japan and Korea (Kwon 1993; Saito et al. 2000).

Kwon (1993) proposed a definition for *Mongoloniscus* based on the following morphological traits: (1) triangular median lobe of cephalon, frontal line separated from vertex by a groove; (2) granulated dorsum, numerous gland pores along the whole margin of pereonites; (3) noduli laterales more or less at the same distance from lateral margin; (4) pereonite 1 evenly convex with postero-lateral corners rounded; (5) pleopodal exopods 1–5 with *Protracheoniscus*-type pseudotrachea; (6) male pleopod 1 exopod with bilobed distal part, and male pleopod 2 endopod with a filiform distal part. However, some species have parts of the above characters that have been assigned to *Mongoloniscus*, leading to a heterogeneous grouping (Kashani 2014; Yang and An 2021).

Recently, the integrative methods of morphology combined with DNA data shed light on taxa delimitation in Oniscidea systematics (Dimitriou et al. 2019). Molecular phylogenetics has revealed numerous cryptic Oniscidea species. Dimitriou et al. (2023) revealed cryptic Armadillidiidae diversity within Cyprus island and discovered two new species. Zimmermann et al. (2018) integrated morphology and molecular analyses to delimit Philosciidae species and reveal a new genus from Brazil. This approach has also been applied to other oniscidean isopods and discovered species of *Hemilepistus*, and *Protracheoniscus* (Gongalsky et al 2018; Wang et al. 2022b). In this study, to objectively identifies *Mongoloniscus* species, we studied the genus from China by integrating morphological characters and molecular data.

## ﻿Materials and methods

**Figure 1.**
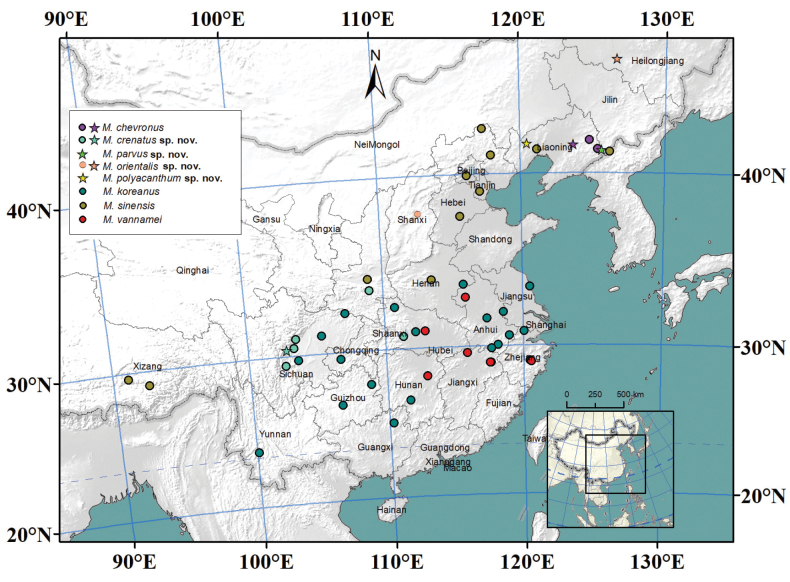
Collected localities of species of *Mongoloniscus* in China. Stars represent type localities.

The specimens were collected at 48 localities of China (Fig. 1), and preserved in 75% ethanol. All specimens were deposited at the Herbarium, National Resource Center for Chinese Materia Medica, China Academy of Chinese Medical Sciences, China (**CMMI**).

### ﻿Morphology

The whole body of the specimens was placed in acid-fuchsin staining buffer for twelve hours. The appendages were dissected and mounted on micro preparations in a neutral balsam mounting medium using a Leica M205 FA microscope. The morphological terminology followed Kwon (1993) and Kwon and Taiti (1993). The taxonomic characters were observed with a Leica M205 FA microscope. Habitus were taken with a Leica M205 MCA camera attached to the microscope. The line drawings were drawn by the GNU Image Manipulation Program (Montesanto 2015).

### ﻿DNA extraction and fragment amplification

Genomic DNA was extracted from tissue samples of each specimen using the Promega Wizard® SV Genomic DNA Purification Kit (Promega, USA). Polymerase chain reaction (PCR) was used to amplify cytochrome *c* oxidase subunit I (COI) and nuclear ribosomal DNA 28S fragments. The COI fragments were amplified using primers LCO1490 and HCOoutout (Folmer et al. 1994; Schwendinger and Giribet 2005), 28S fragments were amplified using primers 28Sa and 28Sb (Michael et al. 1997; Joshi and Karanth 2011) followed the procedure described by Chen et al. (2023). All sequences were deposited in GenBank database, with accession numbers listed in Suppl. material 1: table S1.

### ﻿Molecular analyses

The COI sequences and 28S sequences obtained from this study, as well as data from previous phylogenetic studies obtained from GenBank, were incorporated into the phylogenetic analysis (Poulakakis and Sfenthourakis 2008; Tanaka and Karasawa 2016; Gongalsky et al. 2021; Yang and An 2021; Dimitriou and Sfenthourakis 2022; accession numbers provided as Suppl. material 1: table S1).

Phylogenetic trees were constructed using Bayesian inference (BI) and maximum likelihood (ML) methods, with branch support assessed using standard statistical tests, including bootstrap support (BS) and posterior probability (PP). ML analysis was performed using the IQ-TREE 1.6.8 software tool (Minh et al. 2020) on the PhyloSuite 1.2.3 platform (Zhang et al. 2020) with 250,000 ultrafast bootstraps (Hoang et al. 2018). The evolutionary model for COI was selected under the Akaike information criterion using ModelFinder (Kalyaanamoorthy et al. 2017), GTR+F+I+G4 using Bayesian inference (BI) and TIM+F+I+G4 for maximum likelihood (ML) analyses. BI analyses were conducted using MrBayes 3.2.6 (Ronquist et al. 2012) over 10,000,000 generations, sampled every 1000 generations, with 25% of trees set aside as burn-in. Stationarity was assessed using a split frequency of less than 0.001, and a consensus tree was constructed from the remaining trees. The consensus tree is shown in FigTree 1.4.3 (Rambaut 2016). Several species of *Desertoniscus*, *Hemilepistus*, *Koreoniscus*, *Lucasioides*, *Orthometopon*, and *Protracheoniscus* were included in phylogenetic reconstructions, while *Armadillidiumnasatum* Budde-Lund, 1885 was chosen as the outgroup (accession numbers provided as Suppl. material 1: table S1).

Furthermore, pairwise Kimura 2-parameter distances of the COI sequences between *Mongoloniscus* species were calculated by MEGA X (Kumar et al. 2018).

### ﻿Distribution mapping

The distribution map was made with ArcMap 10.7.1. We illustrated all the collected localities based on the *Mongoloniscus* specimens in the present research.

## ﻿Results

The specimens collected from China were analyzed using external traits and dissected appendages. As a result, eight members of *Mongoloniscus* were preliminarily recognized, including four known species (*M.koreanus*, *M.sinensis*, *M.vannamei* and *M.chevronus*). Among them, the morphological characters of *M.vannamei* and *M.chevronus* indicate that these two species are more likely to belong to *Lucasioides* and *Koreoniscus*, respectively. It is difficult to verify the taxonomic identities based on traditional morphology.

### ﻿Molecular analyses

**Figure 2.**
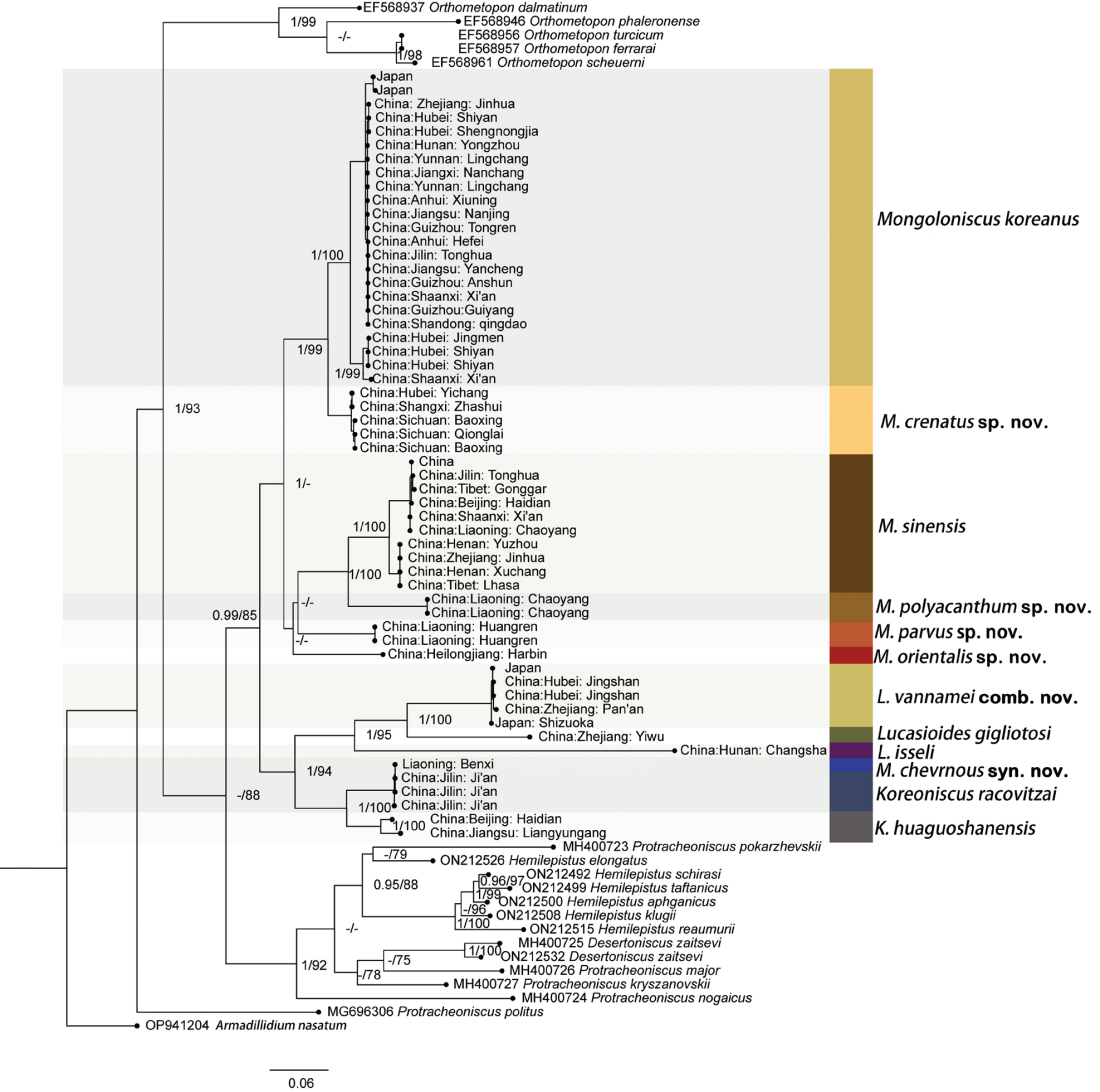
Maximum likelihood phylogenetic tree based on combined COI and 28S data for *Mongoloniscus*. Values above the branches represent the posterior probability (PP) and bootstrap support (BS), BS < 70% or PP < 0.9 are indicated as hyphens.

This study involved the sequencing and alignment of mitochondrial COI and nuclear 28S rRNA loci from *Mongoloniscus* species. Sequences from other Agnaridae genera were also included. A final alignment dataset comprising 793 base pairs (bp) of COI and 741 bp of 28S rRNA was obtained. Maximum likelihood and Bayesian methodologies were employed for phylogenetic analysis (Fig. 2). The results indicated that *M.chevronus* and *Koreoniscusracovitzai* formed a clade with high support (PP = 1.00, BS = 100%), and they were sister species to *K.huaguoshanensis* with high support from both likelihood bootstraps (PP = 1.00, BS = 100%). This suggests that *M.chevronus* is a member of *Koreoniscus*, which is supported by morphological characteristics described below. Furthermore, *M.vannamei*, *L.gigliotosi*, and *L.isseli* formed a clade with high support (PP = 1.00, BS = 95%), indicating that *M.vannamei* must be transferred to *Lucasioides* (see below for details in the taxonomic section).

The remaining *Mongoloniscus* species, including *M.koreanus*, *M.crenatus* sp. nov., *M.sinensis*, *M.polyacanthum* sp. nov., *M.parvus* sp. nov., and *M.orientalis* sp. nov. formed a clade with strong support (PP = 0.99, BS = 85%). Notably, *M.crenatus* sp. nov. was found to be a sister to *M.koreanus* with very high support (PP = 1.00, BS = 99%). The two aforementioned species, both found in southern China, formed a clade that is sister to species distributed in northern China (*M.polyacanthum* sp. nov., *M.parvus* sp. nov., and *M.orientalis* sp. nov.) or in northern China and Tibet (*M.sinensis*). However, this relationship had moderately low support (PP = 1.00, BS = 50%). *M.koreanus* can be divided into two genotypes with high node support (PP = 1.00, BS = 100%), one widespread in southern China while the other is limited to Hubei Province (these two populations are morphologically indistinguishable and are considered intraspecific variations). Similarly, *M.sinensis* was divided into two well-supported clades with no clear morphological or geographic correlations. Thus, they were considered to belong to the same species. In summary, *Mongoloniscus* in China appears to be a monophyletic taxon which is closely related to *Koreoniscus* and *Lucasioides*.

### ﻿Genetic distances

Among *Mongoloniscus* species, the average K2P genetic distance of the COI sequences was 14.0%, the intraspecific distances were 0−3%, and the interspecific distances varied from 10% (*M.crenatus* sp. nov. and *M.koreanus*) to 24% (*M.polyacanthum* sp. nov. and *M.crenatus* sp. nov.) (Suppl. material 1: table S2). Although *M.crenatus* sp. nov. and *M.koreanus* had the lowest genetic distances, the intraspecific genetic distances were still lower than the interspecific distances. The genetic distance between *M.chevronus* and *K.racovitzai* was ~ 0%, showing no difference in the result of the phylogenetic analysis. Thus, we proposed a new synonym (see below for details under the taxonomic section of *K.racovitzai*).

### ﻿Taxonomy

**Order Isopoda Latreille, 1817**

**Suborder Oniscidea Latreille, 1802**

**Family Agnaridae Schmidt, 2003**

#### ﻿Genus Mongoloniscus

Taxon classificationAnimaliaIsopodaAgnaridae

Verhoeff, 1930

B3A60F3D-7951-584F-A7E5-B564B5DD5B57

##### Type species.

*Mongoloniscuskoreanus* Verhoeff, 1930, by subsequent designation.

##### Notes.

**Figure 3.**
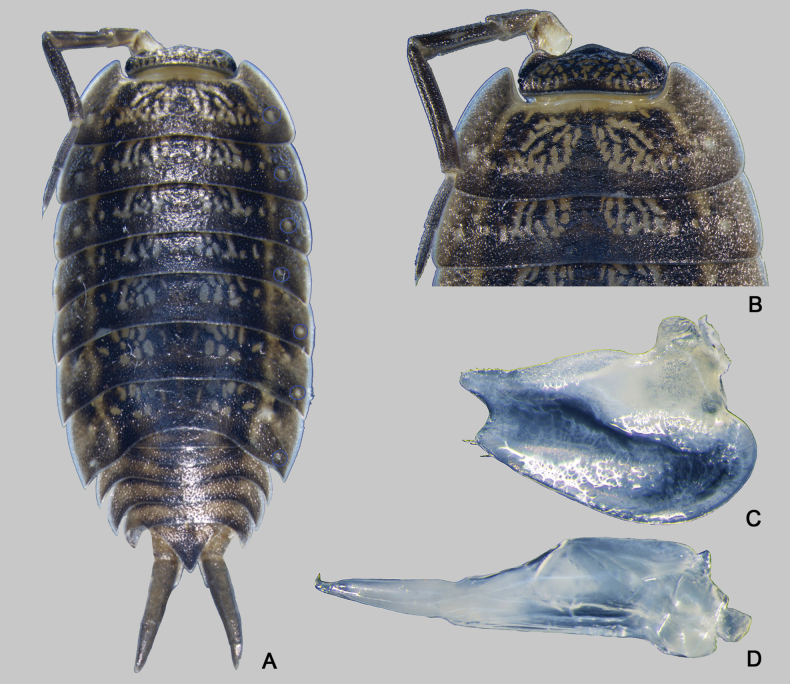
Habitus of *Mongoloniscussinensis* (Dollfus, 1901), male **A** dorsal view, the blue circles show the noduli laterales arrangement **B** cephalon and pereonites 1 and 2 in dorsal view **C** pleopod 1 exopod **D** pleopod 1 endopod.

Previous studies have ascribed eighteen species to the genus *Mongoloniscus*; however, some do not conform to the criteria proposed by Kwon (1993). In the present study, we provide the typical habitus of the genus (Fig. 3) and adopt a more restrictive interpretation, asserting that a species can be classified as *Mongoloniscus* if exhibiting all the following characters:

1. Dorsum granulated, with numerous gland pores along the entire margin of the pereonites.
2. Noduli laterales more or less at the same distance from lateral margin, with a d/c value less than 0.75.
3. Cephalon with well-developed median lobe and lateral lobes, median one convex or arched on anterior margin.
4. Pereonite 1 posterior margin straight or rounded.
5. Pleopodal exopods 1–5 with monospiracular internal lungs.
6. Pleopod 1 exopod with bilobed apex, and posterior tip of endopod bent outwards.

#### ﻿ Mongoloniscus crenatus

Taxon classificationAnimaliaIsopodaAgnaridae

Jiang, Li & Huang sp. nov.

EC0C4DA8-298F-51E8-8C64-DC5864647036

https://zoobank.org/19F87F63-688E-4C59-887E-B78B81A8DF24

Figs 4A, B , 5 , 6

##### Type material.

***Holotype*. China**: ♂ (20210417001), **Sichuan Province**, Baoxing County, Muping Town, Lengmugou Provincial Geological Park (30.3699°N, 102.8125°E), 1020 m asl., 14.iv.2021, coll. Chao Jiang.

***Paratypes*. China**: 3 ♂♂, 8 ♀♀ (20210417002–20210417012), same data as the holotype. 4 ♂♂, 6 ♀♀ (20210412063–20210412066), **Hubei Province**, Yichang, Xiaoxita Forestry Park (30.7853°N, 111.3180°E), 100 m asl., 12.iv.2021, coll. Zhidong Wang & Tianyun Chen. 1 ♂ (20210512006), **Shaanxi Province**, Zhashui County, Lengbinggou Village (33.6931°N, 109.0239°E), 1020 m asl., 12.x.2021, coll. Chao Jiang. 3 ♂♂, 1 ♀ (20210417016–20210417019), **Sichuan Province**, Baoxing County, Dengchigou (30.5341°N, 102.9410°E), 1810 m asl., 17.iv.2021, coll. Chao Jiang; 3♂♂, 1♀ (20210416040–20210416043), Qionglai, Datong Village, Dalong Road (30.5042°N, 103.3063°E), 770 m als., 14.iv.2021, coll. Chao Jiang; 5 ♂♂, 24 ♀♀ (120220827008–20220827025), Hanyuan County, Fuling Town, Hanyuan Service Area (29.4370°N, 102.6343°E), 1080 m asl., 27.viii.2022, coll. Chao Jiang; 6 ♂♂, 9 ♀♀ (20220828036–20220828048), Tianquan County, Binhe Park (30.0584°N, 102.7597°E), 760 m asl., 28.viii.2022, coll. Chao Jiang; 18 ♂♂, 23 ♀♀ (20220829013–20220829028), Wenchuan County, Yingxiu Town (31.0557°N, 103.4887°E), 880 m asl., 29.viii.2022, coll. Chao Jiang.

##### Diagnosis.

Cephalon with median lobe convex, medially with a small incision. Antennal flagellum with distal article twice as long as proximal article. Noduli laterales almost at same distance from the lateral margins. Pereopod 6 basis and pereopod 7 ischium fringed with long setae. Pleopod 1 exopod deeply bilobed at apex, inner lobe much longer than outer lobe; apex of male pleopod 1 endopod bent outwards and pointed.

##### Description.

**Figure 4.**
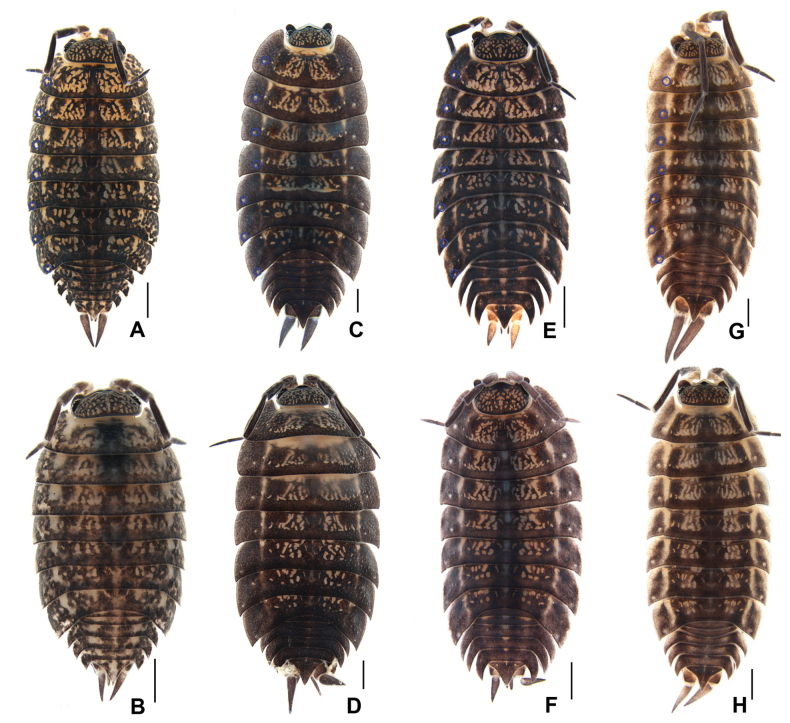
Habitus of *Mongoloniscus* species **A, C, E, G** male, holotype **B, D, F, H** female, paratype **A, B***M.crenatus* sp. nov. **C, D***M.polyacanthum* sp. nov. **E, F***M.parvus* sp. nov. **G, H***M.orientalis* sp. nov. The blue circles show the noduli laterales arrangement. Scale bars: 3 mm.

**Figure 5.**
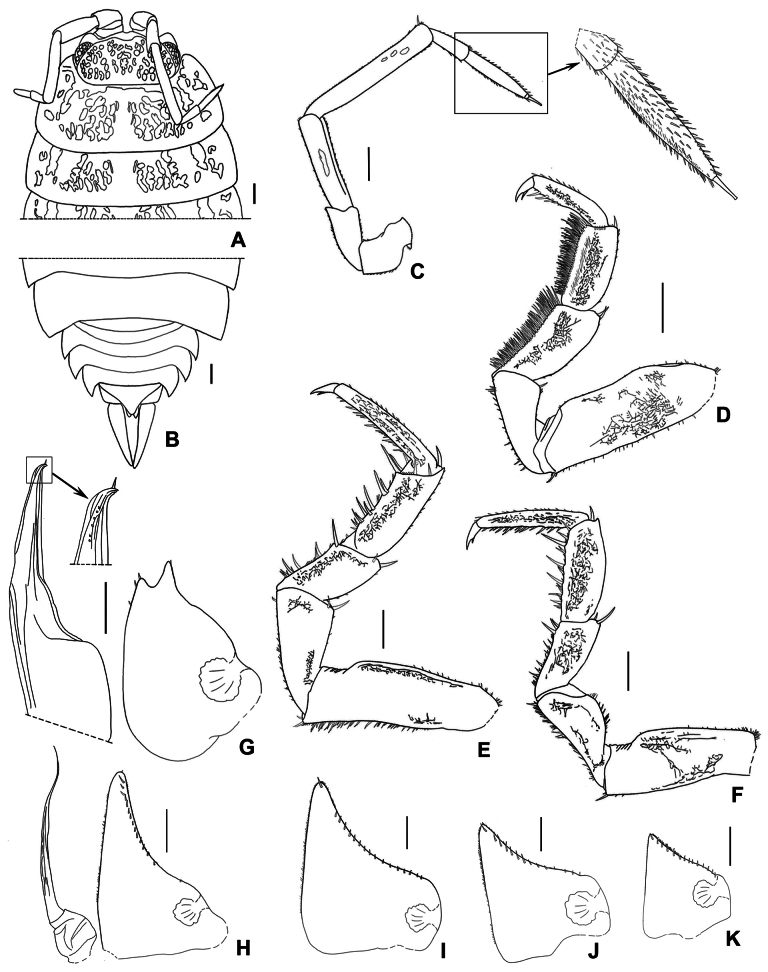
*Mongoloniscuscrenatus* sp. nov., holotype **A** cephalon, pereonites 1 and 2 in dorsal view **B** pleonites, telson and uropod in dorsal view **C** second antenna **D** pereopod 1 **E** pereopod 6 **F** pereopod 7 **G** pleopod 1 **H** pleopod 2 **I–K** pleopods 3–5 exopods. Scale bars: 1 mm.

***Body*** length of males 7–9 mm and females 8–11 mm. Body elongated and convex, ~ 2–2.5× as long as widest pereonite. Dorsum distinctly granulated, brown-gray color with usual yellowish muscle spots. Numerous gland pores along entire pereonite margin. Pereonite 1 with rounded postero-lateral corner, posterior margin nearly straight. Noduli laterales almost at the same distance from lateral margins (Fig. 4A, B). Telson triangular, approximately twice as wide as long, lateral margins slightly concave at distal one third, posterior apex blunted; uropodal exopod ~ 2–2.5 and 1.5–2× as long as protopod in males and females, respectively; protopod with an incision on outer margin (Figs 4A, B, 5B).

***Cephalon*** with rounded lateral lobe no evidence surpasses eyes, median lobe convex, medially with a small incision (Fig. 5A). Eyes with 15–19 ommatidia. Antenna with fifth article of peduncle longer than flagellum; flagellum with distal article 1.6–2.1× as long as proximal one (Fig. 5C).

***Pereopod*** 1 bearing brush of long setae on its carpus and merus (Fig. 5D). Pereopod 6 basis fringed with long setae and a distal protrusion on sternal margin (Fig. 5E). Pereopod 7 ischium with sternal margin slightly concave and fringed with setae, rostral surface with shallow depression; carpus slightly expanded on the tergal margin (Fig. 5F).

***Pleopods*** 1–5 exopods with monospiracular internal lungs (Fig. 5G–K). Male: pleopod 1 exopod oval, with robust sinuous outer margin; apex deeply bilobed and bearing several setae, inner lobe much longer than outer lobe (Fig. 5G). Pleopod 2 exopod nearly triangular, bearing one line of setae on the outer margin (Fig. 4H). Pleopod 1 endopod with broad basal part, narrowed towards apex, apex bent outwards and pointed (Fig. 5G); pleopod 2 endopod longer than exopod, distal article thin and long (Fig. 5H).

##### Remarks.

The new species resembles *M.koreanus* in morphology. It can be distinguished from the latter by the cephalon median lobe medially with a small incision, pereopod 7 with an unexpanded carpus on the tergal margin, and pleopod 1 endopod with a pointed distal apex. In *M.koreanus*, the cephalon median lobe is medially continuous, pereopod 7 has an expanded carpus on the tergal margin, and the distal apex of the pleopod 1 endopod is blunted. Furthermore, this species and *M.koreanus* formed two clades with high support in the phylogenetic analysis (Fig. 2), and their interspecific distances were much higher than their intraspecies distances (Suppl. material 1: table S2).

##### Etymology.

Latin *crenatus* = notched. The new species name refers to the anterior margin of the cephalon with a small notch in the middle. We suggest the Chinese common name as “刻痕蒙潮虫”.

##### Distribution.

China (Hubei, Shaanxi, Sichuan).

#### ﻿ Mongoloniscus polyacanthum

Taxon classificationAnimaliaIsopodaAgnaridae

Jiang, Li & Huang sp. nov.

0945D901-0E1A-5B41-AD10-7DFC5BCDC630

https://zoobank.org/40D39285-4EF1-4344-B62B-FE91BC37D75E

Figs 4C, D , 6

##### Type material.

***Holotype*. China**: ♂ (20210908003), **Liaoning Province**, Chaoyang, Fenghuangshan National Forestry Park (41.54725°N, 120.4743°E), 210 m asl., 8.iv.2021, coll. Chao Jiang.

***Paratypes*.** 1 ♂, 8 ♀♀ (20210908001, 20210908002, 20210908004–20210908010) same data as the holotype.

##### Diagnosis.

Antennal flagellum with distal article ~ 1.2× as long as proximal article. Pereopod 7 ischium sternal margin fringed sparse setae. Pleopod 1 exopod with one line of setae on inner margin, distal apex deeply bilobed, inner lobe as long as outer one, but slightly wider than outer lobe.

##### Description.

**Figure 6.**
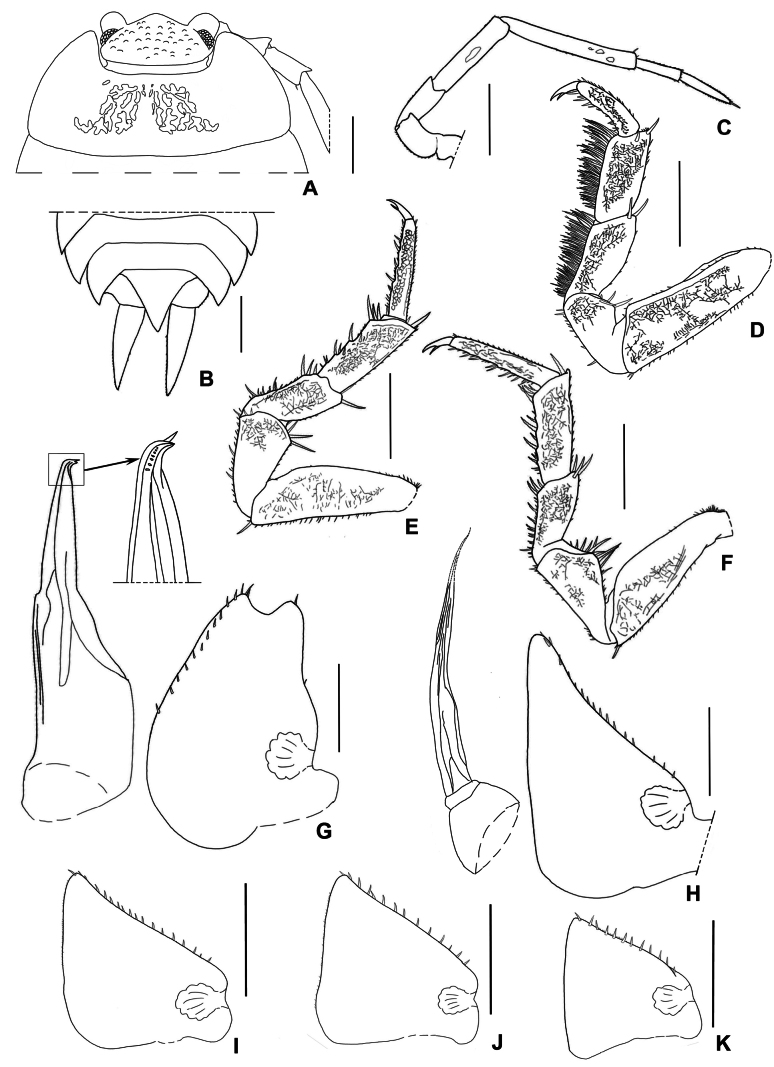
*Mongoloniscuspolyacanthum* sp. nov., holotype **A** cephalon and pereonite 1 in dorsal view **B** pleonites, telson, and uropod in dorsal view **C** second antenna **D** pereopod 1 **E** pereopod 6 **F** pereopod 7 **G** pleopod 1 **H** pleopod 2 **I–K** pleopods 3–5 exopods. The abrupt tip of pleopod 2 endopod are indicated as dotted lines. Scale bars: 1 mm (**A–F**); 0.5 mm (**G–K**).

***Body*** length of males 7–9 mm and females 8–11 mm. Body elongated and convex, ~ 2–2.5× as long as widest pereonite. Dorsum distinctly granulated, brown-gray in color with usual yellowish muscle spots. Numerous gland pores along entire pereonites margin. Pereonite 1 with rounded postero-lateral corner, posterior margin nearly straight. Noduli laterales on pereonites 1–4 much farther from lateral margins than those on pereonites 5–7. Telson triangular, with slightly concave on lateral margins, posterior apex pointed; uropodal exopod ~ 1.5–2× as long as protopod; protopod with an incision on the outer margin (Figs 4C, D, 6B).

***Cephalon*** with medial lobe triangular, not surpassing the lateral lobes in dorsal view (Fig. 6A). Eyes with 20 ommatidia. Antenna with fifth article of peduncle longer than flagellum; flagellum with distal article 1.2× as long as proximal one (Fig. 6C).

***Pereopod*** 1 bearing a brush of long setae on its carpus and merus (Fig. 6D). Pereopod 7 ischium rostral surface with a shallow depression; carpus not expanded on the tergal margin (Fig. 6F).

***Pleopods*** 1–5 exopods with monospiracular internal lungs (Fig. 6G–K). Male: pleopod 1 exopod oval, apex deeply bilobed, inner lobe as long as outer one, but slightly wider than outer lobe; outer margin sinuous, bearing one seta near middle and apex, respectively; inner margin with one line of setae; (Fig. 6G). Pleopod 2 exopod almost triangular, bearing one line of setae on the outer margin. Pleopod 1 endopod with broad basal part, narrowed towards apex, apex acute and bent outwards, bearing several setae (Fig. 6G); pleopod 2 endopod longer than exopod, distal article thin and long (Fig. 6H).

##### Remarks.

This new species is similar to *M.sinensis* in the large body size, noduli laterales arrangement, and the fringed sparse setae on the pereopod 6 basis and pereopod 7 ischium. However, it can be distinguished from the latter by the distal article of the antennal flagellum longer than the proximal article, carpus of pereopod 7 without a rounded lobe on the tergal margin, and pleopod 1 exopod inner margin bearing a line of well-developed setae. In *M.sinensis*, the antennal flagellum articles are equal in length, the carpus of pereopod 7 has a rounded lobe on the tergal margin, and the pleopod 1 exopod bearing 2–4 setae on the apex of the inner lobe. Furthermore, this species together with *M.sinensis* formed two clades with high support in the phylogenetic analysis (Fig. 2), whose interspecific distances were much higher than their intraspecies distances (Suppl. material 1: table S2).

##### Etymology.

Latin prefix *poly*- = many, plus *acanthum* = spinous. The new species name refers to the setae on the inner margin of pleopod 1 exopod. We suggest the Chinese common name as “多刺蒙潮虫”.

##### Distribution.

China (Liaoning).

#### ﻿ Mongoloniscus parvus

Taxon classificationAnimaliaIsopodaAgnaridae

Jiang, Li & Huang sp. nov.

3F4A7951-D3FA-593F-900B-906FC0E7CEC3

https://zoobank.org/E2A83DFA-2E8F-4BC6-8586-105B10A9A34A

Figs 4E, F , 7

##### Type material.

***Holotype*. China**: ♂ (20200913004), **Liaoning Province**, Huanren Manchu Autonomous County: Erpengdianzi Town, Yaoqianshu Village (41.1893°N, 125.6383°E), 610 m asl., 13.ix.2020, coll. Chao Jiang.

***Paratypes*.** 3 ♂♂, 2 ♀♀ (20200913003, 2020091300005), same data as the holotype; 3♂♂, 2♀♀ (20200906002–20200906005), **Liaoning Province**, Huanren Manchu Autonomous County, Erpengdianzi Town (41.236°N, 125.6047°E), 560 m asl., 6.ix.2021, coll. Chao Jiang. 1 ♂, 2 ♀♀ (20200913001, 20200913002), **Jilin Province**, Ji’an, Xihulugou Village (41.3348°N, 125.8893°E), 670 m asl., 13.ix.2020, coll. Chao Jiang.

##### Diagnosis.

Antennal flagellum distal article approximately twice as long as the proximal article. Pereopod 7 ischium sternal margin fringed with sparse setae; carpus slightly expands on tergal margin. Pleopod 1 exopod oval, distal apex slightly concave, forming two inconspicuous lobes, inner lobe bearing one seta.

##### Description.

**Figure 7.**
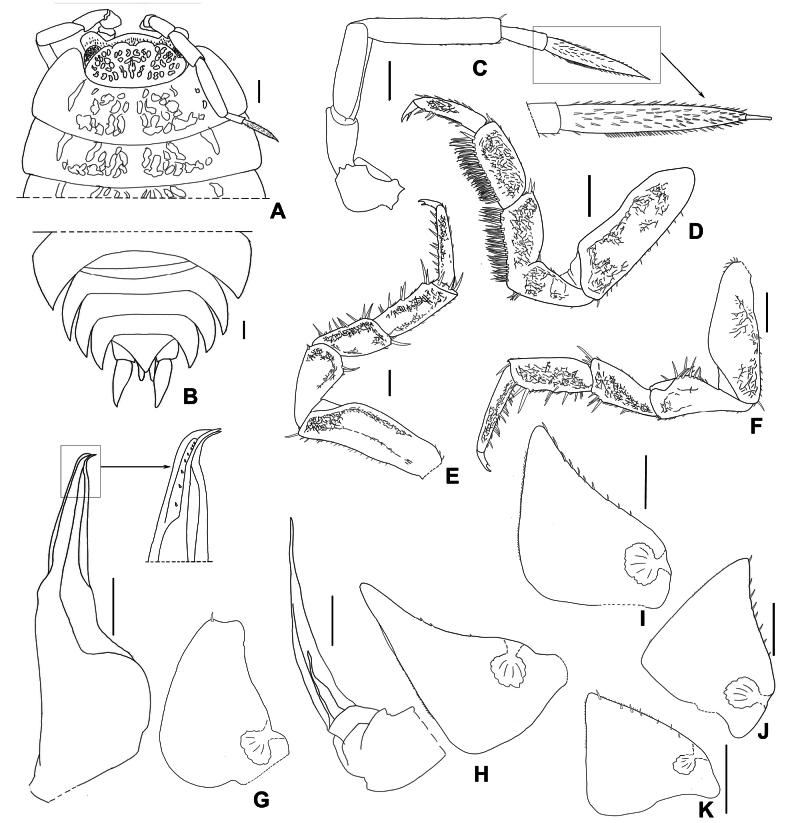
*Mongoloniscusparvus* sp. nov., holotype **A** cephalon, pereonites 1 and 2 in dorsal view **B** pleonites, telson and uropod in dorsal view **C** second antenna **D** pereopod 1 **E** pereopod 6 **F** pereopod 7 **G** pleopod 1 **H** pleopod 2 **I–K** pleopods 3–5 exopods. Scale bars: 1 mm.

***Body*** length of males 5–9 mm and females 4–9 mm. Body elongated and convex, ~ 2.3× as long as widest pereonite. Dorsum distinctly granulated, brown-gray color with usual yellowish muscle spots. Numerous gland pores along entire margin pereonites margin (Fig. 4E, F). Pereonite 1 with rounded posterior margin and postero-lateral corners. Noduli laterales on pereonites 1–4 much farther from lateral margins than those on pereonites 5–7. Telson triangular, ~ 1.5× as wide as long, lateral margins slightly concave near middle, posterior apex blunted; uropodal exopod 1.2–1.5× as long as protopod; protopod with an incision on outer margin (Figs 3F, 4E, 7B).

***Cephalon*** with medial lobe arched, not surpassing lateral lobes in dorsal view. Eyes with 14 or 15 ommatidia. Antenna with fifth article of peduncle and flagellum nearly equal in length; flagellum with distal article twice as long as proximal one (Fig. 7C).

***Pereopod*** 1 bearing a brush of long setae on its carpus and merus (Fig. 7D). Pereopod 7 ischium rostral surface with shallow depression; carpus slightly expands on tergal margin (Fig. 7F).

***Pleopods*** 1–5 exopods with monospiracular internal lungs (Fig. 7G–K). Male: pleopod 1 exopod oval, outer margin sinuous, distal apex slightly concave and forming two inconspicuous lobes, inner lobe bearing one seta near apex (Fig. 7G). Pleopod 2 exopod almost triangular, slightly concave on outer margin (Fig. 7H). Pleopod 1 endopod with broad basal part, narrowed towards apical apex, apex bearing several setae, bent outwards and ending with two pointed tips (Fig. 7G); pleopod 2 endopod longer than exopod, distal article thin and long (Fig. 7H).

##### Etymology.

Latin *parvus* = small. The new species name refers to the pleopod 1 with a small exopod. We suggest the Chinese common name as “小蒙潮虫”.

##### Distribution.

China (Liaoning, Jilin).

#### ﻿ Mongoloniscus orientalis

Taxon classificationAnimaliaIsopodaAgnaridae

Jiang, Li & Huang sp. nov.

A82F149B-6F30-506F-A72E-0F946E46DB0E

https://zoobank.org/B8C37716-798D-4BEA-8A20-F80F39EEE983

Figs 4G, H , 8

##### Type material.

***Holotype*. China**: ♂ (20230403006), **Heilongjiang Province**, Harbin: Xiangfang District, Longrui Residential (126.6821°N, 45.7233°E), 160 m asl., 3.ix.2023, coll. Junjie Zong.

***Paratypes*.** 6 ♂♂, 13 ♀♀ (20230403003–20230403008), same data as the holotype. **China**: 2 ♂♂, 2 ♀♀ (20231030301, -02), **Shanxi Province**, Taiyuan: Longcheng Forestry Park (37.9228°N, 112.7565°E), 1610 m asl., 30.x.2023, coll. Tianyun Chen, Yuan Xiong & Jiabo Fan.

##### Diagnosis.

Antennal flagellum with distal article as long as proximal article. Pereopod 6 basis fringed with long setae. Pereopod 7 ischium with sternal margin slightly concave and fringed with setae carpus with rounded lamellar lobe on tergal margin. Apex of pleopod 1 exopod bilobed, outer lobe larger than inner one.

##### Description.

**Figure 8.**
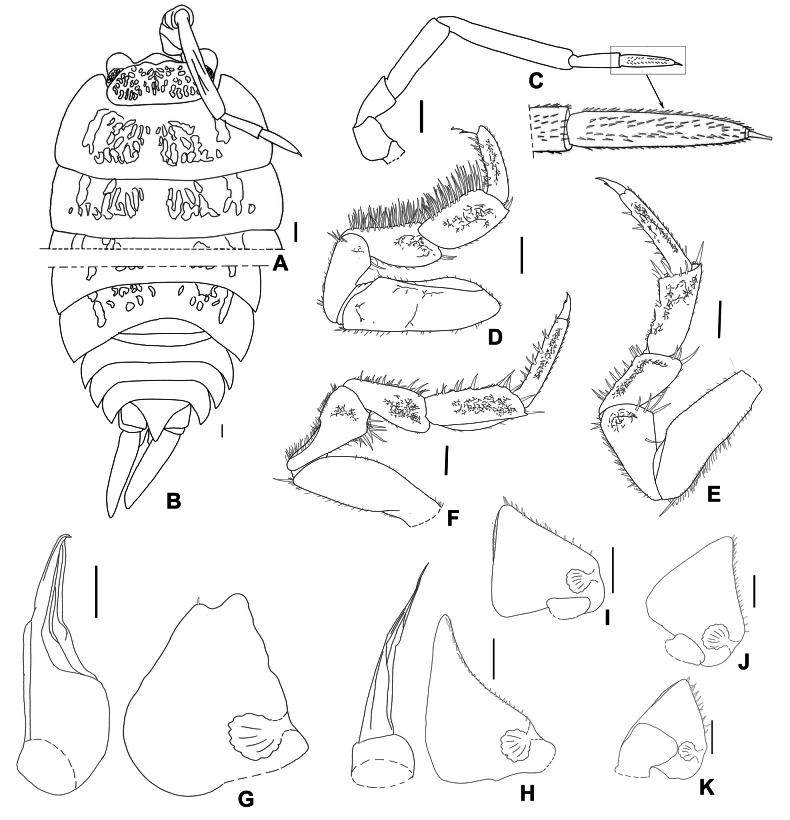
*Mongoloniscusorientalis* sp. nov., holotype **A** cephalon, pereonites 1 and 2 in dorsal view **B** pleonites, telson and uropod in dorsal view **C** second antenna **D** pereopod 1 **E** pereopod 6 **F** pereopod 7 **G** pleopod 1 **H** pleopod 2 **I–K** pleopods 3–5 exopods. Scale bars: 1 mm.

***Body*** length of males 8–12 mm and females 7–16 mm. Body elongated and convex, ~ 2.8× as long as widest pereonite. Dorsum distinctly granulated, brown-gray color with usual yellowish muscle spots. Numerous gland pores along entire pereonites margin (Fig. 4G, H). Pereonite 1 with rounded postero-lateral corners, distal margin nearly straight. Noduli laterales on pereonites 1–4 and 7 shifted from lateral margins than those on pereonites 5 and 6. Telson triangular, slightly wider than length, outer margin slightly concave near middle, posterior apex pointed; uropodal exopod ~ 2.8–3.6× as long as protopod in males and ~ 1.2–2× in females; protopod with an incision on outer margin (Figs 4G, H, 8B).

***Cephalon*** with medial lobe triangular, not surpassing lateral lobes in dorsal view. Eyes with 20 ommatidia. Antenna with fifth article of peduncle longer than flagellum; flagellum with distal article as long as proximal one (Fig. 8C).

***Pereopod*** 1 bearing a brush of long setae on its carpus and merus (Fig. 8D). Pereopod 6 basis fringed with long setae (Fig. 8E). Pereopod 7 ischium with sternal margin slightly concave and fringed with setae, rostral surface with shallow depression; carpus with rounded lamellar lobe on tergal margin (Fig. 8F).

***Pleopods*** 1–5 exopods with monospiracular internal lungs (Fig. 8G–K). Male: pleopod 1 exopod drop-like, outer margin sinuous, apex bilobed; outer lobe larger than inner one, inner lobe bearing one seta at apex (Fig. 8G). Pleopod 2 exopod nearly triangular, bearing one line of setae on outer margin (Fig. 8H). Pleopod 1 endopod with broad basal part, narrowed towards apex, apex bent outwards and pointed (Fig. 8G); pleopod 2 endopod longer than exopod, distal article thin and long (Fig. 8H).

##### Remarks.

This new species resembles *M.koreanus* by basis of pereopod 6 having long setae and a distal protrusion on the sternal margin, ischium of pereopod 7 fringed with setae. However, it can be differentiated from the latter by antenna with two equal flagellum articles, and its noduli laterales on pereonites 1–4 and 7 are much farther from lateral margins than those on pereonites 5 and 6. In *M.koreanus*, the distal article of the flagellum is twice as long as the proximal article, and the noduli laterals are almost at the same distance from the lateral margin. Furthermore, this species together with *M.koreanus* formed two clearly different clades in the phylogenetic analysis (Fig. 2), whose interspecific distances were much higher than their intraspecies distances (Suppl. material 1: table S2).

##### Etymology.

Latin *orientalis* = east. The new species name refers to its distribution in east China. We suggest the Chinese common name as “东方蒙潮虫”.

##### Distribution.

China (Heilongjiang, Shanxi).

#### ﻿ Mongoloniscus koreanus

Taxon classificationAnimaliaIsopodaAgnaridae

(Verhoeff, 1930)

F759144B-9079-5DB4-8A1B-9BBA3245C6CF

Protracheoniscus (Mongoloniscus) koreanus Verhoeff, 1930: 117, figs 14, 15.
Mongoloniscus koreanus : Kwon 1993: 149, figs 12, 13; Kwon 1995: 528.
Mongoloniscus nigrogranulatus Kwon & Taiti, 1993: 48, figs 201–208.
Nagurus tsushimaensis Nunomura, 1987: 30, fig. 113.
Nagurus pallidus : Nunomura 1991: 8, fig. 171.

##### Examined material.

**China**: 2 spms (20200813002, 20200813006), **Anhui Province**, Hefei, Binghu National Forestry Park (31.7230°N, 117.3728°E), 20 m asl., 13.viii.2020, coll. Chao Jiang; 2 spms (20230324029–20230324030), Qimen county: Qishan Town, Qichang Road (29.8666°N, 117.6841°E), 150 m asl., 24.iii.2023, coll. Chao Jiang; 28 spms (20230327116–20230327129), Ningguo County, Yunti She Village, Qianqiushezu (29.8666°N, 117.6841°E), 150 m asl., 27.iii.2023, coll. Chao Jiang; 26 spms (20230326076–20230326088, 20230327102–20230327103), Xijin Streets, Cuizhu Park (30.6319°N, 118.9710°E), 60 m asl., 27–28.iii.2023, coll. Chao Jiang; 9 spms (20230324017–20230324021), Xiuning County, Qiyunshan Service Area (29.7540°N, 118.1321°E), 200 m asl., 24.iii.2023, coll. Chao Jiang; 1 spm (20230325071), Huangshan, Tankou Town, Hougu (30.0740°N, 118.1527°E), 510 m asl., 25.iii.2023, coll. Chao Jiang; 12 spms (20230215006–20230215008), Bozhou: Bozhou Cultural Park (33.8286°N, 115.7616°E), 15.ii.2023, coll. Chao Jiang; 2 spms (20230214004, 20230214005), Fuyang, Yingquan district, Wuming Service Area, (33.0458°N, 115.8935°E), 14.ii.2023, coll. Chao Jiang. 2 spms (20201018028, 20201018037), **Chongqing**, Jiangbei District, Tieshanping Forestry Park (29.5957°N, 106.6645°E), 450 m asl., 18.x.2020, coll. Chao Jiang & Zhidong Wang. 13 spms (20201118001, -12, -18, -20, -22, -25, -30, -37, -38, -40, -41, -43), **Guangxi Zhuang Autonomous Region**, Guilin, Guangxi Guilin National Forestry Park (25.2207°N, 110.2543°E), 18.xi.2020, coll. Zhidong Wang. **Guizhou Province**, 20 spms, (20201020001, -4, -7, -9, -10, -14, -16, -17, -20, -21, -24, -25, -27, -29, -30, -31, -35, -36, -39, -40), Zhengning County, Yelangdong Scenic Area (26.0918°N, 105.6248°E), 1070 m asl., 20.x.2020, coll. Chao Jiang & Zhidong Wang; 8 spms (20210601038–20210601045), Guiyang: Jinhua town, Shangcheng West Road (26.6067°N, 106.57463°E), 1260 m asl., 1.vi.2021, coll. Chao Jiang; 16 spms (20210728020–20210728035), Jiangkou county, Fanjingshan National Nature Reserve (27.8464°N, 108.7733°E), 1510 m asl., 28.vii.2021, coll. Zhidong Wang. 3 spms (20210410069–20210410071), **Hubei Province**, Jingmen, Xiangshan II Road, Youyuan (31.0441°N, 112.1999°E), 80 m asl., 10.iv.2021, coll. Zhidong Wang & Tianyun Chen; 5 spms (20210414063, -64, 20210414066–20210414068), Shennongjia: shennongding (31.4902°N, 110.3583°E), 1840 m asl., 14.iv.2021, coll. Zhidong Wang & Tianyun Chen; 9 spms (20210415051–20210415059), Shiyan, Niutoushan National Forestry Park (32.6118°N, 110.7298°E), 390 m asl., 15.iv.2021, coll. Zhidong Wang & Tianyun Chen; 9 spms (20210415061–20210415069), Yuanyuan Park (32.6113°N, 110.7695°E), 270 m asl., 15.iv.2021, coll. Zhidong Wang & Tianyun Chen; 2 spms (20190321002, 20190321007), **Hunan Province**, Yongzhou, Puliqiao Town (26.6849°N, 111.5997°E), 160 m asl., 21.iii.2019, coll. Chao Jiang; 37 spms (20201107001, -2, -6, -7, -10, -14, -16, -18, -22, -24–-30, -32, -33, -35–-38, -40, -42, -44, -45, -47, -48, -50, -52–-54, -57, -60, -63, -64, -67), **Jiangsu Province**, Yancheng: Jiangsu Yancheng Wetland National Nature Reserve (Rare Birds) (33.6035°N, 120.5042°E), 5 m asl., 7.xi.2020, coll. Zhidong Wang; 9 spms (20201109001, -6, -9, -18, -27, -30, -31, -39, -42), Nanjing, Jiangjunshan Scenic Area (32.1007°N, 118.5861°E), 130 m asl., 9.xi.2020, coll. Zhidong Wang; 2 spms (20210801001, -2), **Jiangxi Province**, Nanchang, Zhaoxian Town, Meiling, (28.7243°N, 115.6848°E), 350 m asl., 1.viii.2021, coll. Zhidong Wang; 32 spms (20230217013–18, -26, -27), Dexing, Yincheng Street, Jishuihu Wetland Park (28.9354°N, 117.5952°E), 70 m asl., 17.ii.2022, coll. Chao Jiang; 23 spms (20230219018–20230219020), Dexing Railway Station (28.9587°N, 117.8544°E), 19.ii.2023, coll. Chao Jiang; 1 spm (20210330053), **Shaanxi Province**, Xi’an, Xi’anbei Railway Station, (34.3741°N, 108.9345°E), 350 m asl., 3.iii.2021, coll. Zhidong Wang; 26 spms (20220909001–20220909026), **Sichuan Province**, Yanting County, Yanting Service Area (31.1481°N, 105.3804°E), 360 m asl., 9.ix.2022, coll. Chao Jiang; 29 spms (20220911007), Tongjiang County: Nuoshuihe Town (32.4320°N, 107.1858°E), 780 m asl., 11.ix.2022, coll. Chao Jiang; 28 spms (20220914002–20220914022), Bazhong, Bazhou District, Jiangbei Road, (31.8718°N, 106.7379°E), 380 m asl., 14.ix.2022, coll. Chao Jiang; 16 spms (20220829070, -71, -73–-84), Meishan, Taihe Town, Meishan Service Area, (31.8718°N, 106.7379°E), 380 m asl., 29.viii.2022, coll. Chao Jiang; 17 spms (20220829045, -47–-60), Jiajiang County, Jiajiang Bridge (29.7216°N, 103.5754°E), 380 m asl., 29.viii.2022, coll. Chao Jiang; 4 spms (20210528013–20210528015, -18), **Yunnan Province**, Linchang, Manpan Street, (23.9061°N, 100.0985°E), 1430 m asl., 28.v.2021, coll. Chao Jiang; 2 spms (20210531016, -17), Mianning Street (23.8787°N, 100.0983°E), 1430 m asl., 31.v.2021, coll. Chao Jiang; 6 spms (20210506004–20210506009), **Zhejiang Province**, Jinhua, Anwen Street, (29.0169°N, 120.4570°E), 390 m asl., 6.v.2021, coll. Chao Jiang; 27 spms (20230324076–20230324086), Hangzhou, Lin’an Service Area, (30.2076°N, 119.5308°E), 160 m asl., 24.iii.2023, coll. Chao Jiang; 2 spms (20230327058), Tianmushan National Nature Reserve (30.3190°N, 119.4448°E), 350 m asl., 27.iii.2023, coll. Chao Jiang; 11 spms (20230328039–20230328044), Huzhou, Huzhou Railway Station (30.8628°N, 120.0238°E), 160 m asl., 28.iii.2023, coll. Chao Jiang; 8 spms (20230324004–20230324009), Wuxing District, Zhihe Road (120.0211E, 30.8706N), 20 m asl., 24.iii.2023, coll. Chao Jiang; 12 spms (20230328057–20230328059), Wuxing District, Renhuangshan Mountain, (30.8973°N, 120.0572°E), 80 m asl., 28.iii.2023, coll. Chao Jiang; 4 spms (20230328008–20230328011), Anji County, Fenghuanshan Park (30.6198°N, 119.7013°E), 160 m asl., 28.iii.2023, coll. Chao Jiang.

##### Remarks.

This species closely resembles *M.maculatus* (Iwamoto, 1943) by the noduli laterals at almost the same distance from the lateral margins, and the morphology of pleopod 1 exopod. However, it can be distinguished in having the eyes with 20–24 ommatidia, male pereopod 6 fringed with long setae and bearing a distal protrusion on the sternal margin at the basis, and the ischium of pereopod 7 fringed with setae as well. In *M.maculatus*, its eyes have 15 or 16 ommatidia, the basis of pereopod 6 and the ischium of male pereopod 7 with sparse setae. For detailed descriptions and illustrations of *M.koreanus* see Kwon (1993).

##### Distribution.

China (Anhui, Chongqing, Guangxi, Guizhou, Hubei, Hunan, Jiangsu, Jiangxi, Shaanxi, Sichuan, Yunnan, Zhejiang), Japan, and Korea.

#### ﻿ Mongoloniscus sinensis

Taxon classificationAnimaliaIsopodaAgnaridae

(Dollfus, 1901)

BFAE302A-3DF5-566A-8801-66B35B5B402B

Metoponorthus (Mongoloniscus) sinensis Dollfus, 1901: 371–374.
Porcellio (Porcellionides) asiaticus : Arcangeli 1927: 175–178.
Mongoloniscus sinensis : Chen 1993: 260.

##### Examined material.

**China**: 1 spm (20180901101), **Beijing**, Haidian District, Tiancun, 1.ix.2018, coll. Junduo Zhang; 1 spm (20230408020), **Hebei Province**, Chengde, Weichuang Manchu and Mongolian Autonomous County, Saihanba National Forestry Park (42.408°N, 117.254°E), 1500 m asl., coll. Tianyun Chen, Yangyang Pan & Jiabo Fan; 8 spms (20230330021–2023033004), Hengshui, Taocheng District, near Hengshui Railway Station (37.7467°N, 115.6913°E), 30.iii.2023, coll. Chao Jiang; 12 spms, 20201212013–20201212018,**Henan Province**, Yuzhou, Xiayu Park (34.1387°N, 113.4801°E), 90 m asl., 12.xii.2020, coll. Chao Jiang; 4 spms (20200912009, 20200913002, -3, -5), **Jilin Province**, Ji’an, Koguryo Archaeological Site Park (41.1210°N, 126.1845°E), 210 m asl., 12–13.ix.2020, coll. Chao Jiang; 14 spms (20210908011–20210908024), **Liaoning Province**, Chaoyang, Fenghuangshan National Forestry Park (41.54725°N, 120.4743°E), 210 m asl., 8.iv.2021, coll. Chao Jiang; 15 spms (20210907005–20210907018), Shangzhi Park (41.5891°N, 120.4302°E), 180 m asl., 7.iv.2021, coll. Chao Jiang; 9 spms (20210904019–20210904026), Huanren Manchu Autonomous County, Zhangyue Park (41.2600°N, 125.3480°E), 270 m asl., 04.ix.2021, coll. Chao Jiang; 8 spms (20210904001, -2, -3, -5, -6, -8, -9), Liaoyang, Baita District, Liaoning Research Institute of Cash Crops (41.2605°N, 121.1392°E), 40 m asl., 4.ix.2021, coll. Chao Jiang; 4 spms (20210907030–20210907032), Xinbin Manchu Autonomous County, Yongling Town (124.7979°E, 41.7193°N), 310 m asl., 4.iv.2021, coll. Chao Jiang, 3 spms (20210907036–20210907038), Nanzamu Town (41.9422°N, 124.4398°E), 210 m asl., 4.iv.2021, coll. Chao Jiang; 27 spms (20210330042–20210330052, 20210330054–20210330069), **Shaanxi Province**, Xi’an, Xi’anbei Railway Station (34.3741°N, 108.9345°E), 350 m asl., 3.iii.2021, coll. Zhidong Wang. 1 spm (20211007001), **Tianjin**, Nancuiping Park (39.0738°N, 117.1483°E), 7.x.2021, coll. Chao Jiang; 25 spms (20210707014–20210707038), **Tibet Autonomous Region**, Lhasa, Nanshan Park (29.6315°N, 91.1146°E), 3670 m asl., 7.vii.2021, coll. Chao Jiang; 7 spms (20210710012–20210710018), Gonggar County, Jiazhulin Town (29.2885°N, 90.8958°E), 3560 m asl., 7.x.2021, coll. Chao Jiang; 1 spm (20210709006), Gyatsa County, Anrao Town (29.1029°N, 92.6002°E), 3230 m asl., 7.ix.2021, coll. Chao Jiang.

##### Remarks.

Most male specimens displayed a rounded lobe on the pereopod 7 carpus tergal margin, except for three specimens from Beijing and Henan province. *M.sinensis* is close to *M.satsumaensis* (Nunomura, 1987) in terms of noduli laterales’ positions and the morphology of pleopod 1 exopod, but it could differ in the antennal flagellum distal article as long as proximal article rather than 1.5× as long as proximal article.

##### Distribution.

China (Beijing, Hebei, Henan, Jilin, Liaoning, Shaanxi, Tianjin, Tibet).

#### ﻿ Koreoniscus racovitzai

Taxon classificationAnimaliaIsopodaAgnaridae

(Arcangeli, 1927)

2F9481A7-3D79-5D0D-A56B-B0FFD30EA605

Porcellio (Lucasius) Racovitzai Arcangeli, 1927: 228, fig. 7.
Koreoniscus racovitzai : Verhoeff 1937: 421; Flasarova 1972:102–111, figs 24–47.
Koreoniscus Racovitzai : Arcangeli 1952: 301.
Mongoloniscus chevronus Yang & An, 2021: 265–274, figs 1–3. syn. nov.

##### Examined material.

**China**: 19 spms (20200912002–20200912004, -7, 20200913001, -4, 20200914001–20200914003), **Jilin Province**, Ji’an, Koguryo Archaeological Site Park (41.1210°N, 126.1845°E), 210 m asl., 12–14.ix.2020, coll. Chao Jiang; 8 spms (20200912011–20200912016), Daqiangfenggou (40.9276°N, 125.9505°E), 330 m asl., 12.ix.2020, coll. Chao Jiang. 10 spms (20210904027–20210904034), **Liaoning Province**, Huanren Manchu Autonomous County, Zhangyue Park (41.2600°N, 125.3480°E), 270 m asl., 4.ix.2021, coll. Chao Jiang; 1 spm (20210905002), Gucheng Town (41.4764°N, 125.3832°E), 380 m asl., 5.ix.2021, coll. Chao Jiang; 1 spm (20210907033), Xinbin Manchu Autonomous County (41.7193°N, 124.7979°E), 310 m asl., 7.ix.2021, coll. Chao Jiang.

##### Remarks.

In the present study, we identified the above specimens by integrating morphological characters and COI sequences. The results demonstrate that not only their morphological traits were the same as *M.chevronus*, but also the COI sequences were 100% identical to the sequence from the type material of *M.chevronus* (GenBank: MW792415) (Yang and An 2021). Thus, we used these materials to analyze the relationships between *M.chevronus* and the other species.

Morphologically, this species is distinctly differing from other *Mongoloniscus* species by its pereonite epimera with a “convex-concave-convex” margin. It is noteworthy that this trait is an essential diagnostic character of *Koreoniscus*. Based on further comparison of the descriptions and illustrations of *M.chevronus* (Yang and An 2021: figs 1–3) and *K.racovitzai* (Flasarová 1972: figs 24–31; Kwon 1993: fig. 11), in addition to the above results obtained through phylogenetic analyses (the genetic distance between *M.chevronus* and *Koreoniscusracovitzai* was nearly 0%, Suppl. material 1: table S2; *M.chevronus* and *Koreoniscusracovitzai* formed a clade with high support, Fig. 2), and with the external morphology and the coordinates of noduli laterales on pereonites, we consider *M.chevronus* Yang & An, 2021 as junior synonym of *Koreoniscusracovitzai* (Arcangeli, 1927).

##### Distribution.

China (Liaoning, Jilin), Japan, Korea.

#### ﻿ Lucasioides vannamei

Taxon classificationAnimaliaIsopodaAgnaridae

(Arcangeli, 1927) comb. nov.

DE08F3D0-5CA7-51EF-971B-47B536CD5D0F

Porcellio (Nagara) Van Namei Arcangeli, 1927: 243.
Porcellio (Nagara) sundaicus : Arcangeli 1927: 248, fig 15.
Nagara (Nagara) Van Namei: Arcangeli 1952: 302.
Protracheoniscus (Mongoloniscus) nipponicus Arcangeli, 1952: 299.
Mongoloniscus nipponicus : Kwon 1993: 150, figs 14, 15.
Mongoloniscus vannamei : Kwon 1995: 527.

##### Examined material.

**China**: 1 spm (20230214007), **Anhui province**, Fuyang, Yingquan District, Wuming Service Area (33.0458°N, 115.8935°E), 14.ii.2023, coll. Chao Jiang; 50 spms (20230215013–20230215023), Bozhou, Bozhou Cultural Park (33.8286°N, 115.7616°E), 15.ii.2023, coll. Chao Jiang; 10 spms (20230218001–20230218008), **Jiangxi Province**, Dexing, Raoshoukun Park (28.9558°N, 117.5608°E), 18.ii.2023, coll. Chao Jiang; 11 spms (20230219001–20230219008), Dexing Railway Station (28.9587°N, 117.8544°E), 19.ii.2023, coll. Chao Jiang; 15 spms (20230214001–20230214008), Jiujiang, Saiyang Town, near Lushan cable-way Station (29.2926°N, 115.9512°E), 16.ii.2023, coll. Chao Jiang; 32 spms (20230216019–20230216033), Chaisang district, Zhonghuaxianmu Park (29.6144°N, 115.9002°E), 16.ii.2023, coll. Chao Jiang; 9 spms (202302180039, -40), Leping, Hongyan Town, Hongyanxianjing Scenic Area (29.0442°N, 117.4738°E), 18.ii.2023, coll. Chao Jiang; 19 spms (20230218048–20230218054, -56, -57), Gaojia Town (28.9949°N, 117.4425°E), 18.ii.2023, coll. Chao Jiang; 12 spms (20210409088–20210409090), **Hubei Province**, Jingshan County, Kongshandong Scenic Area (30.9728°N, 113.0415°E), 100 m asl., coll. Zhidong Wang & Tianyun Chen; 3 spms (20210410034), Huzhuashan National Forestry Park (31.0765°N, 112.9009°E), 200 m asl., coll. Zhidong Wang & Tianyun Chen. 2 spms (20210508051, -52), **Zhejiang Province**, Pan’an County, Dapanshan Medicinal Plant Garden (28.9827°N, 120.5536°E), 680 m asl., coll. Chao Jiang.

##### Remarks.

*Mongoloniscusvannamei* distinctly differs from all other *Mongoloniscus* species by the noduli laterales on pereonites 2–4 and 7 which are much farther from the lateral margins than those on pereonites 1, 5, and 6. We recognized this species based on the diagnostic characters among the similar genera *Mongoloniscus*, *Lucasioides*, *Agnara*, *Koreoniscus* and *Protracheoniscus* (Kwon 1993), and found that all the traits of *M.vannamei* match the generic characters of *Lucasioides*, except for its epimeron of pereonite 1 was not bent outwards. Furthermore, *M.vannamei*, *L.gigliotosi* and *L.isseli* formed a clade with high support (PP = 1.00, BS = 95%) according to the results of the phylogenetic analysis (Fig. 2). Thus, *M.vannamei* must be transferred to *Lucasioides*. For species descriptions and illustrations, see Kwon (1993).

##### Distribution.

China (Anhui, Hubei, Hunan, Jiangxi, Zhejiang), Japan, Korea.

## ﻿Discussion

Currently, all similar genera within the family Agnaridae are separated by the morphological characters, e.g., *Mongoloniscus* Verhoeff, 1930 can be distinguished from *Lucasioides* Kwon, 1993 by the arrangement of noduli laterals and the shape of the first pereonite (Kwon 1993; Gongalsky et al. 2021). In morphological taxonomy, although eighteen species have been ascribed to the genus *Mongoloniscus* (Boyko et al. 2008), rather than strictly conforming to the criteria proposed by Kwon (1993), several of these species only have some of the diagnostic characters of the genus. These identifications made a dilemma: the species recognition of *Mongoloniscus*, whether based on the only morphological criteria or parts of diagnostic traits, is uncertain.

Considering that the DNA-based approach has revealed an effective way to resolve the taxonomic problems of terrestrial isopods (e.g., Zeng et al. 2021; Khalaji-Pirbalouty et al. 2022; Raupach et al. 2022; Wang et al. 2022a; Yoshino and Kubota 2022), we present mitochondrial COI and nuclear 28S rRNA data based on a broad sample of taxa in the present study (Suppl. material 1: table S1). In the molecular analyses, the results support the use of COI sequences as a useful DNA barcode marker for identifying *Mongoloniscus* species (Suppl. material 1: table S2), and indicate that *Mongoloniscus* is a monophyletic taxon closely related to *Koreoniscus* and *Lucasioides* (Fig. 2). Based on a combination of morphological taxonomy and molecular analyses, we propose *Lucasioidesvannamei* (Arcangeli, 1927), comb. nov. out of *Mongoloniscus*, and *M.chevronus* Yang & An, 2021 as junior synonym of *Koreoniscusracovitzai* (Arcangeli, 1927). Furthermore, we provide a more restrictive interpretation of *Mongoloniscus*, making the generic characters more conducive to future species identifications.

If we follow the principle of the restrictive definition, several species previously categorized as *Mongoloniscus* members may be transferred to the other genera, e.g., *M.persicus* Kashani, 2014, *M.katakurai* (Nunomura, 1987), and *M.vannamei* (Arcangeli, 1927) should be transferred to *Lucasioides* because their noduli laterales on pereonites 2–4 are distinctly shifted from the lateral margins than those on the other pereonites, instead of at nearly the same distance from the lateral margin. Other congeners, such as *M.amabilis* Nunomura, 2013, *M.masahitoi* (Nunomura, 1987), *M.arvus* Nunomura, 2010, *M.hokurikuensis* (Nunomura, 1987), *M.persicus* Kashani, 2014, *M.ishikawai* Nunomura, 2013 and *M.circacaudatus* (Nunomura, 1987) *etc.*, have similar taxonomic problems. These findings highlight the need for the genus to be revised in future studies. In this context, integrative taxonomy could be considered an effective method for resolving taxonomic ambiguities.

## Supplementary Material

XML Treatment for Mongoloniscus

XML Treatment for Mongoloniscus crenatus

XML Treatment for Mongoloniscus polyacanthum

XML Treatment for Mongoloniscus parvus

XML Treatment for Mongoloniscus orientalis

XML Treatment for Mongoloniscus koreanus

XML Treatment for Mongoloniscus sinensis

XML Treatment for Koreoniscus racovitzai

XML Treatment for Lucasioides vannamei

## ﻿Additional information

### Author ORCIDs

Chao Jiang https://orcid.org/0000-0003-1841-1169

Weichun Li https://orcid.org/0000-0003-0154-861X

### Data availability

All of the data that support the findings of this study are available in the main text or Supplementary Information.

## Supplementary materials

Supplementary material 1 Supporting Information Data type docx Explanation note **table S1.** Species vouchers and GenBank accession numbers; **table S2.** Pairwise genetic divergence (K2P-distance) among *Mongoloniscus* species of China using COI sequences. https://binary.pensoft.net/file/1054582This dataset is made available under the Open Database License (http://opendatacommons.org/licenses/odbl/1.0/). The Open Database License (ODbL) is a license agreement intended to allow users to freely share, modify, and use this Dataset while maintaining this same freedom for others, provided that the original source and author(s) are credited.Chao Jiang, Jing Zhong, Zhidong Wang, Weichun Li, Luqi Huang
