# Supplementary material for: ﻿Taxonomic study on the genus Mongoloniscus Verhoeff, 1930 (Isopoda, Agnaridae) from China: morphological and phylogenetic analyses
Source: Zookeys. 2024 May 23;1202:229–53. doi: 10.3897/zookeys.1202.113560 (PMC11140264; doi:10.3897/zookeys.1202.113560)
Supplement: Supplementary material 1 — Supporting Information [file zookeys-1202-229_article-113560__-s001.docx]

**Supporting information**

**Table S1.** Species vouchers and GenBank accession numbers.

| **No.** | **Species** | **Voucher** | **Locality** | **COI** | **28S** | **References** |
| --- | --- | --- | --- | --- | --- | --- |
| 1 | *M. koreanus* | 20201118012 | China: Guangxi: Guilin | OR523703 | OR527959 | This study |
| 2 | *M. koreanus* | 20201107001 | China: Jiangsu: Yancheng | OR523704 | OR527960 | This study |
| 3 | *M. koreanus* | 20210506008 | China: Zhejiang: Jinhua | OR523707 | OR527963 | This study |
| 4 | *M. koreanus* | 20210410070 | China: Hubei: Jingmen | OR523708 | OR527964 | This study |
| 5 | *M. koreanus* | 20210414067 | China: Hubei: Shengnongjia | OR523710 | OR527966 | This study |
| 6 | *M. koreanus* | 20210415051 | China: Hubei: Shiyan | OR523711 | OR527967 | This study |
| 7 | *M. koreanus* | 20210415058 | China: Hubei: Shiyan | OR523712 | OR527968 | This study |
| 8 | *M. koreanus* | 20210415067 | China: Hubei: Shiyan | OR523713 | OR527969 | This study |
| 9 | *M. koreanus* | 20201018028 | China: Chongqing: Jiangbei | OR523714 | OR527970 | This study |
| 10 | *M. koreanus* | 20190321007 | China: Hunan: Yongzhou | OR523715 | OR527971 | This study |
| 11 | *M. koreanus* | 20200813002 | China: Anhui: Hefei | OR523716 | OR527972 | This study |
| 12 | *M. koreanus* | 20201107003 | China: Shandong: qingdao | OR523717 | OR527973 | This study |
| 13 | *M. koreanus* | 20201020016 | China: Guizhou: Anshun | OR523719 | OR527975 | This study |
| 14 | *M. koreanus* | 20210601039 | China: Guizhou:Guiyang | OR523720 | OR527976 | This study |
| 15 | *M. koreanus* | 20210728023 | China: Guizhou: Tongren | OR523721 | OR527977 | This study |
| 16 | *M. koreanus* | 20201109027 | China: Jiangsu: Nanjing | OR523722 | OR527978 | This study |
| 17 | *M. koreanus* | 20210330053 | China: Shaanxi: Xi'an | OR523724 | OR527980 | This study |
| 18 | *M. koreanus* | 20230324017 | China: Anhui: Xiuning | OR523743 | OR527999 | This study |
| 19 | *M. koreanus* | 20210528014 | China: Yunnan: Lingchang | OR523729 | OR527985 | This study |
| 20 | *M. koreanus* | 20210531017 | China: Yunnan: Lingchang | OR523730 | OR527986 | This study |
| 21 | *M. koreanus* | 20210801002 | China: Jiangxi: Nanchang | OR523731 | OR527987 | This study |
| 22 | *M. koreanus* | Ag-1 | Japan | LC017825 | - | Tanaka and Karasawa 2016 |
| 23 | *M. koreanus* | Ag-5 | Japan | LC017826 | LC017821 | Tanaka and Karasawa 2016 |
| 24 | *M. sinensis* | 20210330052 | China: Shaanxi: Xi'an | OR523705 | OR527961 | This study |
| 25 | *M. sinensis* | 20201212015 | China: Henan: Xuchang | OR523706 | OR527962 | This study |
| 26 | *M. sinensis* | 20200912009 | China: Jilin: Tonghua | OR523718 | OR527974 | This study |
| 27 | *M. sinensis* | 20210330051 | China: Shaanxi: Xi'an | OR523723 | OR527979 | This study |
| 28 | *M. sinensis* | 20210707024 | China: Tibet: Lhasa | OR523732 | OR527988 | This study |
| 29 | *M. sinensis* | 20210710015 | China: Tibet: Gonggar | OR523733 | OR527989 | This study |
| 30 | *M. sinensis* | 20210908017 | China: Liaoning: Chaoyang | OR523735 | OR527991 | This study |
| 31 | *M. sinensis* | - | China | MG709492 | - | GenBank |
| 32 | *M. sinensis* | 20201212017 | China: Henan: Yuzhou | OR523739 | OR527995 | This study |
| 33 | *M. sinensis* | 20220724001 | China: Beijing: Haidian | OR523740 | OR527996 | This study |
| 34 | *M. crenatus* sp. nov. | 20210412064 | China: Hubei: Yichang | OR523709 | OR527965 | This study |
| 35 | *M. crenatus* sp. nov. | 20210512006 | China: Shangxi: Zhashui | OR523725 | OR527981 | This study |
| 36 | *M. crenatus* sp. nov. | 20210417009 | China: Sichuan: Baoxing | OR523726 | OR527982 | This study |
| 37 | *M. crenatus* sp. nov. | 20210416041 | China: Sichuan: Qionglai | OR523727 | OR527983 | This study |
| 38 | *M. crenatus* sp. nov. | 20210417017 | China: Sichuan: Baoxing | OR523728 | OR527984 | This study |
| 39 | *M. polyacanthum* sp. nov. | 20210908001 | China: Liaoning: Chaoyang | OR523734 | OR527990 | This study |
| 40 | *M. polyacanthum* sp. nov. | 20210908002 | China: Liaoning: Chaoyang | OR523738 | OR527994 | This study |
| 41 | *M. parvus* sp. nov. | 20210906004 | China: Liaoning: Huangren | OR523741 | OR527997 | This study |
| 42 | *M. parvus* sp. nov. | 20210906003 | China: Liaoning: Huangren | OR523742 | OR527998 | This study |
| 43 | *M. orientalis* sp. nov. | 20230403007 | China: Heilongjiang: Harbin | OR523744 | OR528000 | This study |
| 44 | *M. vannamei* | 20210508051 | China: Zhejiang: Pan'an | OR523746 | OR528002 | This study |
| 45 | *M. vannamei* | 20210410034 | China: Hubei: Jingshan | OR523747 | OR528003 | This study |
| 46 | *M. vannamei* | 20210409089 | China: Hubei: Jingshan | OR523748 | OR528004 | This study |
| 47 | *M. vannamei* | Ag-20 | Japan: Fukuoka | LC017827 | LC017822 | Tanaka and Karasawa 2016 |
| 48 | *M. vannamei* | wa3436_Che561 | Japan: Shizuoka | LC496519 | - | GenBank |
| 49 | *M. chevronus* | - | China: Liaoning: Benxi | MW792415 |  | Yang and An 2021 |
| 50 | *Lucasioides isseli* | 20211006063 | China: Hunan: Changsha | OR523749 | OR528005 | This study |
| 51 | *L. gigliotosi* | 20210510020 | China: Zhejiang: Yiwu | OR523750 | OR528006 | This study |
| 52 | *Koreoniscus racovitzai* | 20200912004 | China: Jilin: Ji'an | OR523736 | OR527992 | This study |
| 53 | *K. racovitzai* | 20200914001 | China: Jilin: Ji'an | OR523737 | OR527993 | This study |
| 54 | *K. racovitzai* | 20200912016 | Jilin: Ji'an | OR523745 | OR528001 | This study |
| 55 | *K. huaguoshanensis* | 20201106016 | China: Jiangsu: Liangyungang | OR523751 | OR528007 | This study |
| 56 | *K. huaguoshanensis* | 20210403028 | China: Beijing: Haidian | OR523752 | OR528008 | This study |
| 57 | *Hemilepistus aphganicus* | 15h | Iran: Mashhad | ON212500 | - | Dimitriou and Sfenthourakis 2022 |
| 58 | *H. klugii* | 12h | Iran: Komijan | ON212508 | - | Dimitriou and Sfenthourakis 2022 |
| 59 | *H. elongatus* | 32h | Iran: Shahrood | ON212526 | - | Dimitriou and Sfenthourakis 2022 |
| 60 | *H. schirasi* | 4h | Iran: Neyriz | ON212492 | ON312029 | Dimitriou and Sfenthourakis 2022 |
| 61 | *H. reaumurii* | 25h | Tunisia | ON212515 | - | Dimitriou and Sfenthourakis 2022 |
| 62 | *H. taftanicus* | 37h | Iran: Khash sistan | ON212499 | - | Dimitriou and Sfenthourakis 2022 |
| 63 | *Protracheoniscus pokarzhevskii* | Psp_A | Russia: Kalmykia | MH400723 | - | Gongalsky et al. 2018 |
| 64 | *P. nogaicus* | Pnog_B | Russia: Kalmykia | MH400724 | - | Gongalsky et al. 2018 |
| 65 | *P. major* | Pmjr_D | Russia: Kalmykia | MH400726 | - | Gongalsky et al. 2018 |
| 66 | *P. politus* | A | Hungary: Budapest | MG696252 | - | Gongalsky et al. 2018 |
| 67 | *P. kryszanovskii* | Pkrz_E | Russia: Kalmykia | MH400727 | - | Gongalsky et al. 2018 |
| 68 | *Desertoniscus zaitsevi* | Dzts_C | Russia: Kalmykia | MH400725 | - | Gongalsky et al. 2018 |
| 69 | *D. zaitsevi* | 77Y | Russia: Bolshoy Tsaryn | ON212532 | - | Dimitriou and Sfenthourakis 2022 |
| 70 | *Orthometopon dalmatinum* | - | Greece: Crete Island | EF568937 | - | Poulakakis and Sfenthourakis 2008 |
| 71 | *O. phaleronense* | - | Greece: Sterea Ellada | EF568946 | - | Poulakakis and Sfenthourakis 2008 |
| 72 | *O. turcicum* | - | Greece: East Aegean | EF568956 | - | Poulakakis and Sfenthourakis 2008 |
| 73 | *O. ferrarai* | - | Greece: East Aegean | EF568957 | - | Poulakakis and Sfenthourakis 2008 |
| 74 | *O. scheuerni* | - | Turkey: Mugla | EF568961 | - | Poulakakis and Sfenthourakis 2008 |
| 75 | *Armadillidium nasatum* | TU-27 | - | OP941204 | - | GenBank |

**Table S2.** Pairwise genetic divergence (K2P-distance) among *Mongoloniscus* species of China using COI sequences.

| Species | 1 | 2 | 3 | 4 | 5 | 6 | 7 | 8 | 9 |
| --- | --- | --- | --- | --- | --- | --- | --- | --- | --- |
| 1 *M. koreanus* | 0.02 |  |  |  |  |  |  |  |  |
| 2 *M. sinensis* | 0.19 | 0.03 |  |  |  |  |  |  |  |
| 3 *M. crenatus* sp. nov. | 0.10 | 0.19 | 0.01 |  |  |  |  |  |  |
| 4 *M. parvus* sp. nov. | 0.15 | 0.18 | 0.17 | 0 |  |  |  |  |  |
| 5 *M. polyacanthum* sp. nov. | 0.23 | 0.19 | 0.24 | 0.20 | 0 |  |  |  |  |
| 6 *M. orientalis* sp. nov. | 0.15 | 0.18 | 0.14 | 0.15 | 0.20 | - |  |  |  |
| 7 *M. chevronus* | 0.16 | 0.18 | 0.17 | 0.17 | 0.22 | 0.13 | - |  |  |
| 8 *Koreoniscus racovitzai* | 0.16 | 0.18 | 0.17 | 0.17 | 0.22 | 0.13 | 0 | 0 |  |
| 9 *Lucasioides vannamei* comb. nov. | 0.23 | 0.24 | 0.24 | 0.23 | 0.23 | 0.19 | 0.21 | 0.21 | 0 |
